# Synthesis and characterization of amino sulfonic acid functionalized graphene oxide as a potential carrier for quercetin

**DOI:** 10.55730/1300-0527.3409

**Published:** 2022-02-23

**Authors:** Ali SADEGHI, Mohammad FAELI, Mahmood TAJBAKHSH, Abdolreza ABRI, Monireh GOLPOUR

**Affiliations:** 1 Department of Chemistry, Islamic Azad University, Jouybar Branch, Jouybar, Iran; 2 Faculty of Chemistry, University of Mazandaran, Babolsar, Iran; 3 Chemistry Department, Azarbaijan Shahid Madani University, Tabriz, Iran; 4 Molecular & Cell Biology Research Center, Faculty of Medicine, Mazandaran University of Medical Science, Sari, Iran

**Keywords:** Nanocomposite, nanocarrier, drug delivery, cytocompatibility

## Abstract

In this investigation, a new nanocomposite as a nanocarrier based on functionalized graphene oxide was synthesized by reaction of tetraethylenepentamine and chlorosulfonic acid with functional groups on the surface of GO. The synthesized nanocomposite was characterized by FT-IR, XRD TGA, FE-SEM, EDAX, TEM, and AFM analysis. To explore the potential of nanocomposites in drug delivery, the loading and releases of quercetin as an anticancer drug were investigated. The result displayed that more than 90% of the drug was loaded on the nanocarrier and the release of the drug is depending on the pH of the environment such that the releases of the drug in Gastric and intestinal conditions were up to 50% and 30%, respectively. The analysis of toxicity effect on the normal and cancer cells indicated that the nanocarrier with the drug has potential for cancer cells therapy without cytotoxic effect on normal cells in IC_50_ concentration.

## 1. Introduction

Research on graphene and graphene oxide (GO) has been widespread and impressive in biomedical applications in new days due to particular properties including two-dimensional flat structure, high mechanical and chemical stability, low toxicity, and good biocompatibility. These properties provide an encouraging method towards designing, advanced drug delivery systems, offer a new class of graphene-based nanomaterial [1–4]. However, as a disadvantage for application, graphene has a high tendency for aggregation duo to interlayers interaction. GO is synthesized from graphene by an oxidation process that produces a versatile material with –COOH, epoxy and –OH functional groups on two sides sheets and edges [5]. These functional groups facilitate exfoliation of GO in different media to create homogeneous solutions that improve the application of GO in different fields such as drug delivery and elimination of the environmental pollutions on the basis of non/covalent bonding e.g., hydrogen bonding and electrostatic interactions [6–10]. Among groups for functionalization, the –NH_2_ is a good candidate with simple chemical modification. Functionalization of reactive surface amine is a convenient method in many studies for the synthesis of modified GO for application in optoelectronic, biodevices, polymer composites, and drug delivery [11–13]. Several groups have focused on the functionalization of GO as a nanocarrier for drug delivery, because of its high potential chemical versatility [14–17]. Zhang et al., for instance, prepared a nanocomposite based on functionalized GO for coloading of camptothecin (CPT) and doxorubicin. Their study displays that the nanocomposite has a high ability for loading of two medicine and good efficiency on the cancer cell compared with pure GO [18].

Quercetin (QCN) is a bioflavonoid that is found in many vegetables and fruits. This natural organic substance exhibits pharmacological properties such as antiinflammatory and anticancer properties due to the absorption of free radicals [19–21]. But, the therapeutic application of this medicine is limited due to some inappropriate properties such as poor solubility in water and low stability and bioavailability. To overcome the mentioned problems, a practical procedure is the use of nanocarriers including GO[22], chitosan, and liposomes [23–26]. There are some studies on the application of nanomaterials for QCN delivery. However, studies on the modified GO as a drug delivery system for QCN are very rare. Accordingly, in the present work, we aim to prepare a modified GO as a nanocarrier with the potential of loading and sustaining release for QCN. In addition, the cytotoxic effect of drug and nanocarrier with the drug compared on human normal and cancer cells. As a novelty, the tetraethylenepentamine and chlorosulfonic were used to functionalize the surface of GO with several hydrogen bonding and active site to make it suitable for proper loading of (QCN) as an anticancer drug.

## 2. Materials and methods

### 2.1. Materials

Graphite, sulfuric acid (98%), phosphoric acid (98%) potassium permanganate, hydrogen peroxide (30 wt.%), hydrochloric acid (38%), ethanol (95%), methanol, acetone, tetraethylenepentamine (TEPA), chlorosulfonic acid, dichloromethane, quercetin (QC), and 3-(4,5-dimethylthiazol-2-yl)-2,5-diphenyltetrazolium bromide (MTT) were purchased from Sigma (USA). Dimethyl sulfoxide was purchased from Merck company. All the reagents utilized in this article had analytical grades and without further purification. The human colorectal adenocarcinoma cell lines (HT29) were bought from the Pasteur Institute, (Tehran, Iran). The human dermal fibroblast cells (HDF) as a normal cell, isolated from human newborn foreskins at 1 month of age that underwent routine circumcision in Amirkola Children’s Hospital (Babol, Iran).

### 2.2. Preparation of GO

A modified Hummers method was used to synthesize GO from natural graphite [27]. In a common procedure, H_2_SO_4_ (120 mL) and H_3_PO_4_ (13 mL) were poured into a three-neck flask then graphite (1 g) was added and stirred at ambient temperature for 24 h. After that KMnO_4_ (7.0 g) was slowly entered into the flask for 70 min at 60 °C. The mixture was stirred for 6 h at 60 °C and then was cooled to less than 5 °C. After that, 200 mL of deionized water and 4 mL of 30% aq H_2_O_2_ were added to it. Yellow sediment was obtained. To remove the remaining salt, sediment was washed with HCl 10% (100 mL) and deionized water (2000 mL). Finally, the solid was separated by centrifuge and dried at 70 °C in an oven for 24 h.

### 2.3. Preparation of GO-TEPA

The mixture of GO (200 mg) and tetraethylenepentamine (3 mL) in ethanol (80 mL 98%) was dispersed by sonication with an ultrasonic apparatus for 80 min and stirring for 48 h at room temperature. Then, nanoparticle suspensions were removed from the solution by filtration, and then washed several times with ethanol, then dried in an oven at 70 °C for 24 h.

### 2.4. Preparation of GO-TEPA-SO_3_H

GO-TEPA (200 mg) was taken in 5 mL of dry dichloromethane in a round bottom flask, then sonicated for 60 min and stirred for 45 min. Subsequently, concentrated chlorosulfonic acid was poured into 15 mL dry dichloromethane and added slowly to the mixture for about 45 min under stirring at 0 °C. After 4h stirring at room temperature, the suspension was filtrated, washed with ethanol and then vacuum dried for 48 h [28–30].

### 2.5. Measurements

Fourier transform infrared (FT-IR) analysis was taken on a Bruker Tensor 27 Spectrometer (Bruker, Karlsruhe, Germany). X-ray diffraction patterns (XRD, Shibuya-ku, Japan) were collected at room temperature on a RigakuD/Max-2550 powder diffractometer by scanning at a rate of 5°/min, in the range of 2 Ɵ = 5–70 °C. The surface morphology of the materials was assessed using scanning electron microscopy (Hitachi S4160 model, Tokyo, Japan) fitted with an energy dispersive X-ray analyzer (EDAX). Using the LENSES STAPT-1000 calorimeter (Germany) under N_2_ atmosphere, thermal gravimetric analysis (TGA) was conducted in the temperature range of 30–800 °C at a heating rate of 10 °C/min. Transmission electron microscopy (TEM) images were obtained using JEOL2100F. Atomic force microscope (AFM) images were recorded using an ICON atomic force microscope (BRUKER) in tapping mode. Raman spectroscopy was taken on the Terkam apparatus equipped with a 532 nm laser and 30mW of power.

### 2.6. Quercetin loading

A hundred mg of GO-TEPA-SO_3_H as a carrier was dispersed in 50 mL of QCN (100 ppm) in ethanol and stirred for 24 h at 25 °C. The solid that contains quercetin loaded on the carrier was separated by centrifuge for 20 min at 3200 rpm and dried in a vacuum desiccator for 24 h. In the following, the amount of unloaded drug in the supernatant was measured with a PG instruments T80 UV-Visible spectrophotometer at 375 nm (λ _max_ of QCN).

### 2.7. Release of QCN from GO-TEPA-SO_3_H

The released values of QCN from GO-TEPA-SO_3_H were measured at 37 °C by dialysis bag at two different pH. The GO-TEPA-SO_3_H-QCN (30 mg) was poured into a phosphate buffer saline solution (PBS) (10 mL, pH 1.2, and 7.4), then it was transferred into the two dialysis bags, and then they were placed in the PBS solution (30 mL) with the similar pH separately under stirring (200 rpm) at 37 °C. After that, a certain volume was taken from the vessel after a specific time, and the same volume of fresh PBS was added to the vessel to ensure a constant total volume. The values of QCN were estimated up to 96 h by a uv–vis spectrophotometer at 375 nm.

### 2.8. Cytotoxicity of nanocomposite (MTT assay)

The cytotoxic effects of the QCN and GO-TEPA-SO_3_-DRUG against HDF human dermal fibroblast cells and HT29 human colorectal adenocarcinoma cell line were determined through the MTT assay. 7 × 10^3^ cells/well HT29 and 1 × 10^4^ cells/well HDF were seeded on a 96-well cell culture plate. The culture media was removed and replaced by a solution containing different concentrations of QCN and GO-TEPA-SO_3_-DRUG (500, 250, 125, and 62.5 μg mL^−1^) after 24 h incubation and cells were incubated for 48 h under standard conditions. The control group was treated with a cell culture medium only. The incubation culture media was removed after 72 h and each well washed with PBS. Fifty mL of MTT (3-(4,5-dimethylthiazol-2-yl)-2,5-diphenyltetrazolium bromide) solution in PBS (5 mg mL^−1^) was entered to each well, and incubated for an additional 4 h. Then, MTT solvent DMSO was added and the absorbance of the solution was recorded at 570 nm (formation of formazan). Finally, the percentage of viability was calculated as the following: ((A 570 nm) sample/((A 570 nm) control) × 100

## 3. Results and discussion

This study was conducted based on presenting a new graphene oxide nanocomposite which can be utilized in the drug delivery system. Graphene oxide as one of the favorable and reliable structures was employed and the structure was modified using the suitable material. The full processes as displayed in Scheme were done using a general synthetic manner and described in detail previously. To assess the synthesized structure and consideration on its capability in loading and releases of the drug; at first, spectral and structural characterizations were done. Then, quercetin as a model drug with the anticancer property was selected and loading, releases and toxicity of the drug on the nanocarrier were studied.

### 3.1. XRD patterns

**Figure 1:**
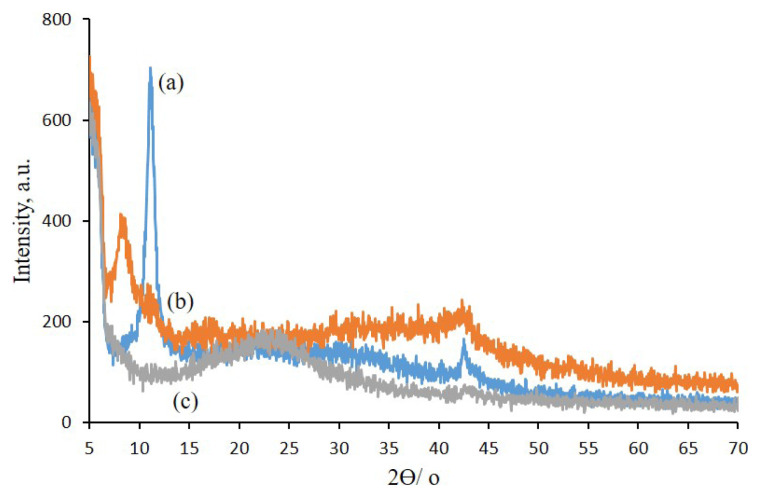
XRD curves of GO (a), GO-TEPA (b), and GO-TEPA-SO_3_H (c).

The energy dispersive X-ray analyzer (XRD) patterns of GO, GO-TEPA, and GO-TEPA-SO_3_H are shown in Figure 1. As a result of abundant oxygen-containing groups, XRD pattern of GO (Figure 1a) shows a sharp peak around the 2θ = 12°. After surface modification functional of GO with TEPA, since more space was created between layers due to the presence of TEPA, the diffraction peak shifts to 2θ = 8° (Figure 1b). The XRD patterns of GO-TEPA-SO_3_H (Figure 1c) compared to GO-TEPA display a peak shift from 2θ = 10° for GO-TEPA to 2θ = 23° for GO-TEPA-SO_3_H, that indicate the exfoliation of GO sheets because of reaction with chlorosulfonic acid. This observation also emphasizes the elimination of functional groups on the surface of GO and reduction of GO [31–32].

### 3.2. Thermal stability

**Figure 2:**
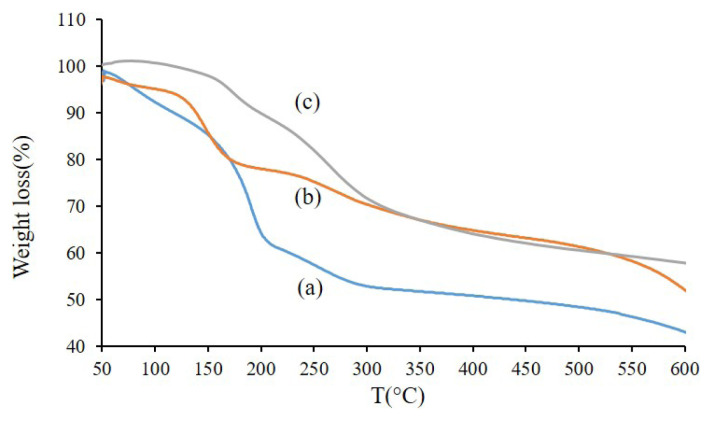
TGA patterns of GO (a), GO-TEPA (b), and GO-TEPA-SO_3_H (c).

The thermal properties of GO, GO-TEPA, and GO-TEPA-SO_3_H were studied using thermal gravimetric analysis (TGA), and the findings are presented in Figure 2. The first weight loss for GO (a) was occurred below 100 °C because of the loss of water. The maximum weight loss for GO (Figure 2a) was observed around 170 °C, which is related to the elimination of oxygen-containing functional groups on the GO. As clear in curve (a), the ultimate weight loss for GO was 56%. In the GO-TEPA curve (Figure 2b), the first weight loss under 150 °C is corresponded to the elimination of water in the structure of the compound, the second happened between 150 °C to 200 °C that assign to the oxygen-containing functional groups, the third mass loss was occurred higher than 200 °C that related to the decomposition of organic functional groups. In the TGA curve of GO-TEPA-SO_3_H (Figure 2c), a first mass loss observed between 50–170 °C was attributed to the water evaporation in the structure of nanocarrier. The second mass loss after 170 °C is related to the destruction of amino sulfonic acid and organic functional groups presented in the GO-TEPA-SO_3_H [30,33–34].

### 3.3. SEM, EDX, TEM, and AFM Analysis

**Figure 3:**
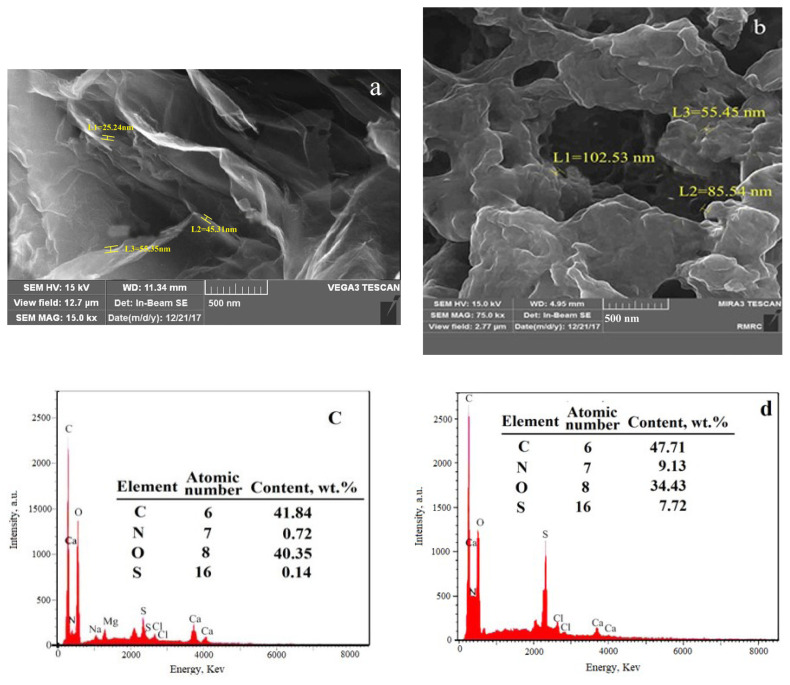
SEM images of GO (a), GO-TEPA-SO_3_H (b) and EDX diagram of GO (c), GO-TEPA-SO_3_H (d).

**Figure 4:**
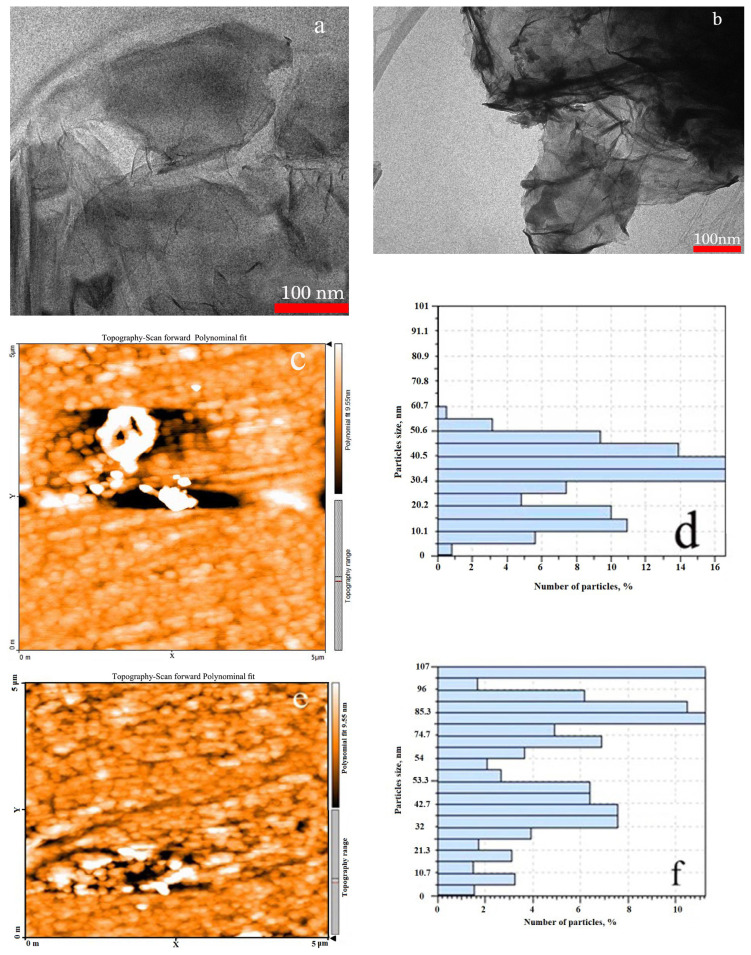
TEM images of GO (a), GO-TEPA-SO_3_H (b) and AFM images of GO (c,d), GO-TEPA-SO_3_H(e,f).

The GO and GO-TEPA-SO_3_H surface morphology was shown by scanning electron microscopy (SEM) images (Figure 3a and Figure 3b). These images indicate that the morphology of GO-TEPA-SO_3_H has been changed compared with GO. The smooth surface and wrinkled edge morphology were observed in the SEM image of GO. These changes have occurred probably from the new functional groups that were fixed on the GO surface [30] Also, from the EDX spectra (Figure 3c and Figure 3d) the presence of (N and S) on GO-TEPA-SO_3_H after the functionalization is clear. Also, the morphology of the GO and GO-TEPA-SO_3_H nanoparticles were studied by transmission electron microscopy (TEM) and atomic force microscopy (AFM) techniques. It is clear from Figure 4b that the TEM image of GO-TEPA-SO_3_H exhibits rough edges compared with that of GO (Figure 4a). This feature may be created after the functionalization of GO surface. Figure 4c, Figure 4d and Figure 4e, Figure 4f displays the AFM images of GO and GO-TEPA-SO3H, respectively. The changes in the size of nanoparticles are evident that proved the probable reaction between GO, amine, and chlorosulphonic acid.

### 3.4. FT-IR of nanomaterial

**Figure 5:**
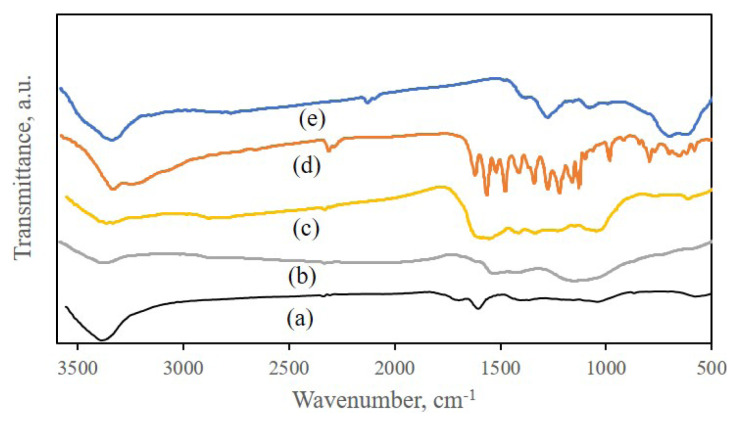
FT-IR spectra of GO(a), GO-TEPA(b), GO-TEPA-SO_3_H(c), QCN(d), and GO-TEPA-SO_3_H-QCN(e).

The Fourier transform infrared (FTIR) spectra of different samples were displayed in Figure 5. The absorption peaks of GO (Figure 5a) at around 3440, 1720, 1628, 1168, 1054, and 875 cm^−1^ are attributed to the stretching vibrations of O-H, C=O, C=C, C-O, and C-O-C symmetric and asymmetric stretching vibrations, respectively. The spectrum of GO-TEPA (Figure 5b) displayed broad peaks related to the NH and OH stretching vibrations above 3400 cm^−1^ that overlaps together. The new peaks were observed between 2800–3000 cm^−1^ that related to CH_2_ stretching vibrations. The NH and CN stretching and bending vibration spectrum were observed at 1554 cm^−1^ and 1434 cm^−1^, respectively. The absorption peaks at 1720 cm^−1^ (C=O stretching), 1054 cm^−1^ (C-O-C symmetric stretching), and 875 cm^−1^ (C-O-C asymmetric stretching) were not observed. These results demonstrated that the tetraethylenepentamine was successfully grafted onto GO [35]. In the spectrum of GO-TEPA-SO_3_H (Figure 5c), the introduction of SO_3_H to the surface of GO-TEPA can be confirmed by such peaks for stretching vibration of N-SO_3_H group at 1615 cm^−1^, asymmetric stretching vibration of SO_3_ at 1358 cm^−1^, and stretching vibration bands at 1247 cm^−1^ and 776 cm^−1^ that assign to S=O and S-N groups, respectively [36–37]. In the spectrum of QCN (Figure 5d) characteristic peaks appeared around 1664 cm^−1^ (C=O stretching), 1610, and 1521 cm^−1^ (peaks of benzene rings). After loading of QCN on the nanocomposite, in the spectrum GO-TEPA-SO_3_H-QCN (Figure 5e) broadband has been observed in areas between 1000–1200 cm^−1^ and 1500–1700 cm^−1^, which are probably due to electrostatic interactions between QCN and nanocomposites and the formation of hydrogen bonding between two compounds.

### 3.5. Raman analysis

**Figure 6:**
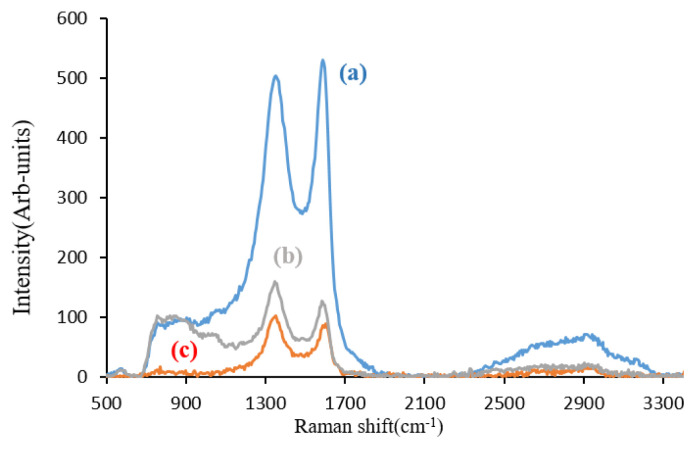
Raman spectra of GO(a), GO-TEPA-SO_3_H(b), and GO-TEPA(c).

The Raman spectra for GO, GO-TEPA, and GO-TEPA-SO_3_H are shown in Figure 6. Raman spectra show two strong peaks at near 1350 nm (D-band) and 1580 nm (G-band). D-band is a reflection of lattice disorder mainly because of the breathing mode of aromatic rings and thus may be used as evidence of defect formed by surface functionalization; while G-band is caused by stretching of the carbon-carbon bond. Therefore, the ratio of intensity of D-band to G-bond (I_D_/I_G_) is an indication of functionalization degree [38–41]. This ratio for GO (Figure 6a) was increased from 0.96 to 1.24 and 1.26 after functionalization to GO-TEPA (Figure 6c) and GO-TEPA-SO_3_H (Figure 6b), respectively. This result indicates that functional groups are attached by covalent bonds to the surface of GO during GO-TEPA and GO-TEPA-SO_3_H synthesis.

## 4. Study of quercetin loading and release from the nanocarrier

### 4.1. Drug loading and release behavior

**Figure 7:**
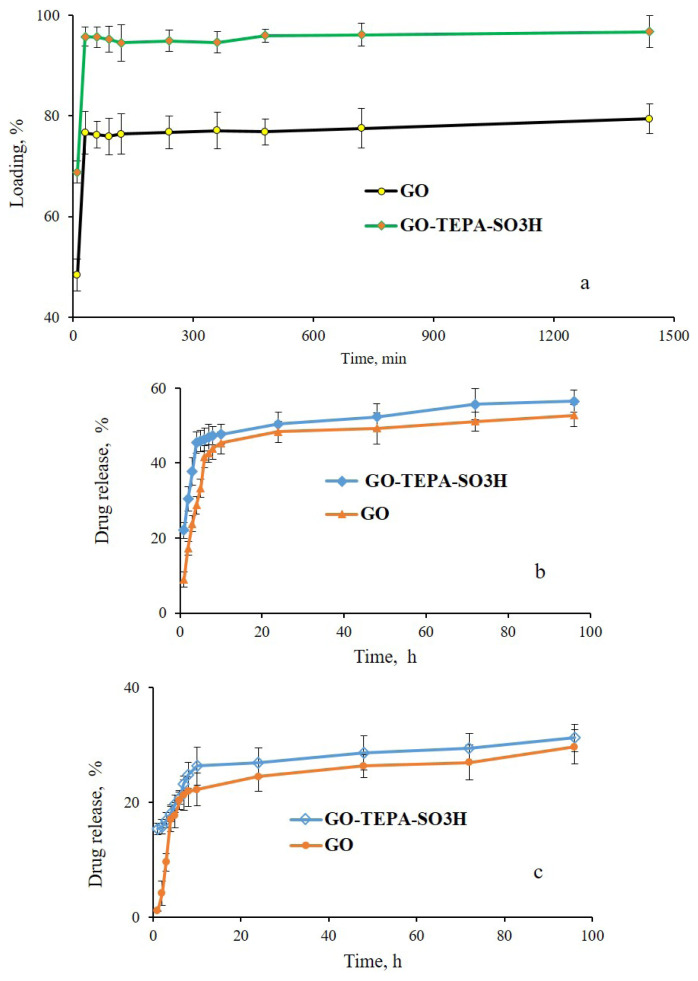
Loading percent of QCN on GO and GO-TEPA-SO_3_H (a). Release profile (b, pH = 1.2 and c, pH = 7.4) of QCN by GO, GO-TEPA-SO_3_H.

Quercetin as a biological material has a special property such as antioxidant and antiinflammatory that is widely used in the formulation of anticancer medicine, but its applied dosage is restricted by some intense side effects. Because of the characteristic and suitable structure, utilizing the GO and modified GO as a carrier of QCN, the side effects of QCN could be effectively reduced. Therefore, QCN is utilized to test the behavior loading and release of GO and GO-TEPA-SO_3_H. With the great ratio of surface to volume, GO and GO-TEPA-SO_3_H are assumed to be able to load quercetin. As shown in Figure 7a, the loading capacity of QCN on GO-TEPA-SO_3_H incremented with the increase connection time and finally reached its saturation value when the initial drug concentration was 100 ppm. The maximum loading capacities of QCN on GO and GO-TEPA-SO_3_H were up to 33.5 and 45.40 mg g^−1^, respectively. This behavior of loading is probably related to the potential of GO and GO-TEPA-SO_3_H to create hydrogen banding and π-π interaction with QCN. The release test of QCN from GO and GO-TEPA-SO_3_H in PBS solution was carried out in pH 1.2 and 7.4 at 37 °C and the results are shown in Figure 7b and Figure 7c, respectively. In the acidic condition (Figure 7b) the total value of drug released during 96 h was about 56.44% and 52.68% for GO-TEPA-SO_3_H and GO, respectively. In an environment with pH 7.4 (Figure 7c), the quantities of drug released were about 31.19% and 29.61% during the 96 h for GO-TEPA-SO_3_H and GO, respectively. The fast QCN release at pH 1.2 was perhaps due to the dissociation of the hydrogen-bonding interaction between the QCN and nanocarrier under acidic environment. Nevertheless, these hydrogen-bonding and π-π stacking interactions between the drug and the nanocarrier are more stable at pH 7.4. Generally, QCN could be loaded on the GO–TEPA-SO_3_H and released more in the acidic condition than basic, typical of micro-environments of cancerous tissues. This feature of nanocarrier provides an ideal mechanism for selective drug release.

### 4.2. Cytotoxicity of QCN and GO-TEPA-SO_3_H-QCN nanomaterial

**Figure 8:**
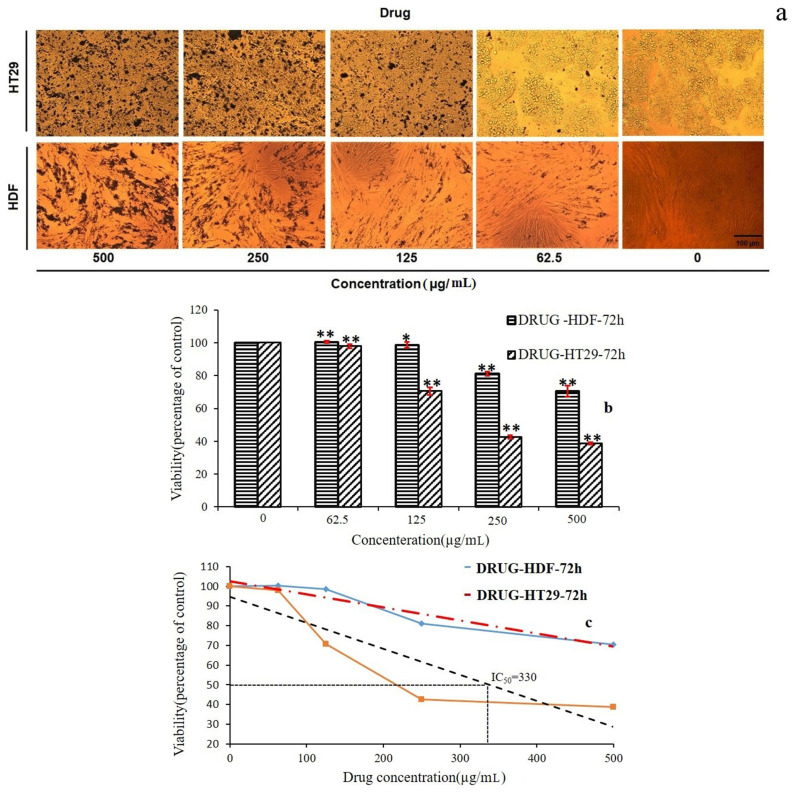
Microscopic images of HT29 and HDF cells after 72 h incubation with different concentrations of QCN (Magnification 20x) (a), Cytotoxic effect of QCN on viability percentage of HT29 and HDF cells (b), IC_50_ value of QCN on HT29 and MDF Cells (c) (* = p < 0.05 and ** = p < 0. 01).

The cytotoxic effect of five concentrations of QCN (0, 62.5, 125, 250, and 500 μg mL^−1^) was evaluated on HT29 human colorectal adenocarcinoma cells and HDF human dermal fibroblast as normal cells using MTT assay. As shown in Figure 8a, Figure 8b, and Figure 8c, in comparison with HDF cells, not only concentrations of QCN have more effect on the viability and morphology but also the increasing of QCN concentration leads to more decreasing in the number of attached cells on HT29 cells. MTT analysis (Figure 8b) on HT29 cell lines shows a decrease in viability rate of cells by increasing the QCN concentration and IC_50_ concentrations of QCN which demonstrated 50% growth inhibition for HT29 cells were 330 μg mL^−1^. While the IC_50_ concentrations of QCN for HDF cells were 500 μg mL^−1^ and there is a significant difference between HT29 cells IC_50_ concentrations (330 μg mL^−1^) and HDF cells IC_50_ concentrations (500 μg mL^−1^) (Figure 8c).

**Figure 9:**
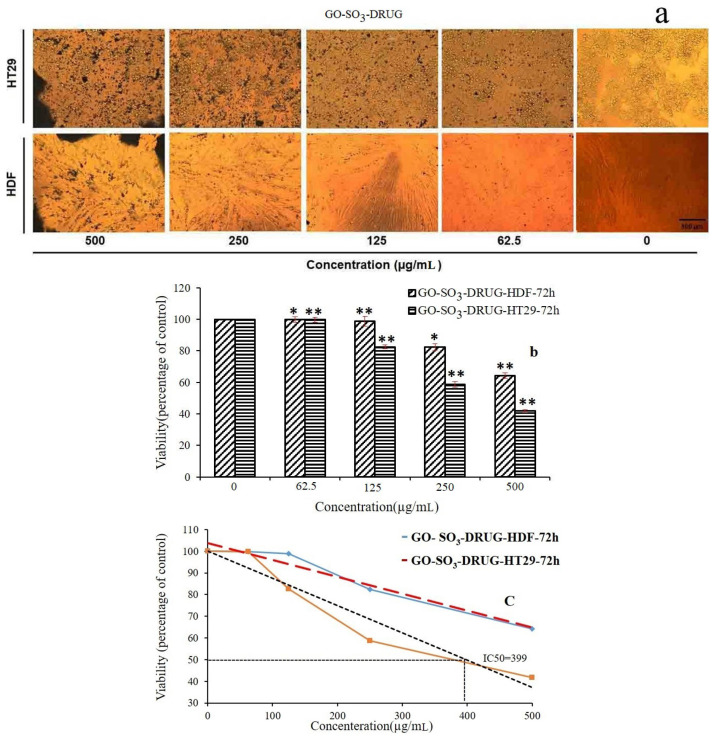
Microscopic images of HT29 and HDF cells after 72 h incubation with different concentrations of GO-TEPA-SO_3_-DRUG (Magnification 20x) (a), Cytotoxic effect of GO-TEPA-SO_3_-DRUG on viability percentages of HT29 and HDF cells (b), IC50 value of GO-TEPA-SO_3_-DRUG on HT29 and HDF Cells (c) (* = p < 0.05 and ** = p < 0.01).

Similarly, Figure 9a shows the same results on the cytotoxic effects of GO-TEPA-SO_3_H-DRUG on HT29 and HDF. A decreased viability rate of cells was indicated by MTT analysis on GO-TEPA-SO_3_H-DRUG treated HT29 cell lines and IC_50_ concentration of GO-TEPA-SO_3_H-DRUG for HT29 cells were 399 μg mL^−1^ but the IC_50_ concentrations of GO-TEPA-SO_3_H-DRUG for HDF cells were 500 μg mL^−1^ and similar to QCN there is significant difference between HT29 cells IC_50_ concentrations (399 μg mL^−1^) and HDF cells IC_50_ concentrations (500 μg mL^−1^) as observed in Figure 9b and Figure 9c. Consequently, we can say, the GO-TEPA-SO_3_H-DRUG has the ability for being used in cancer cells therapy without cytotoxic effect on normal cells in IC_50_ concentration.

## 5. Conclusion

In summary, the tetraethylenepentamine (TEPA) and chlorosulfonic acid-functionalized graphene oxide were successfully prepared from the graphene oxide. It introduced more hydrogen bonding and active sites on the surface of GO to make proper interactions with quercetin in order to optimize the drug loading. FT-IR, XRD, TGA, SEM, EDX, TEM, and AFM spectral characterization documented that the TEPA and chlorosulfonic acid were grafted onto the GO. In the case of initial concentration of QCN at 100 ppm, the nanocarrier influenced an excellent capability of binding anticancer drug QCN with a high loading percentage up to 90%, and also the drug release from the novel delivery can be controlled by pH of environment. GO-TEPA-SO_3_H nanocarrier had faster and more release in acidic pH = 1.2 up to 50% and the release was about 31% in pH = 7.4. Also, MTT assay for QCN and QCN loaded on GO-TEPA-SO_3_H nanocarriers on the HDF and HT29 cells relieved that QCN loaded nanocarrier exhibited more cytotoxicity than free QCN, hence, it can release QCN in cell medium and import more QCN into cancer cells. Therefore, the nanocarrier can be a proper candidate as a new drug delivery system with great capacity in cancer therapy.

## Figures and Tables

**Scheme:**
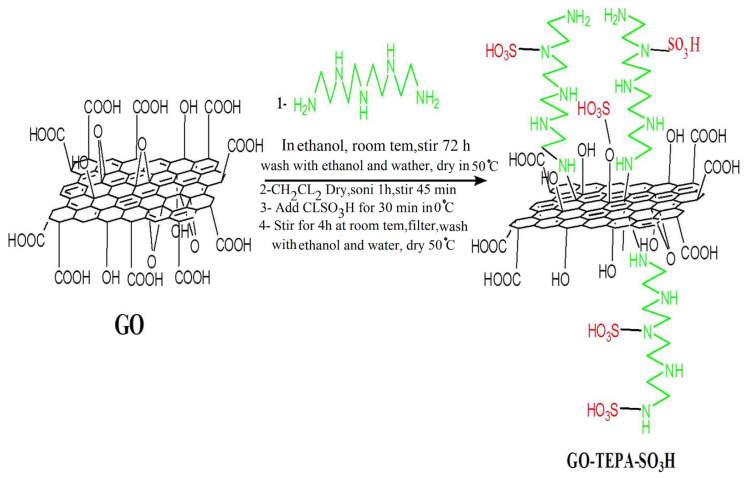
represents the synthetic pathway GO-TEPA-SO_3_H.

## References

[1] Fu Y Chen H Sun X Wang X Combination of cobalt ferrite and graphene: High-performance and recyclable visible-light photocatalysis Applied Catalysis B: Environmental 2012 111–112 280 287 10.1016/j.apcatb.2011.10.009

[2] Lian Q Luo A An Z Li Z Guo Y Au nanoparticles on tryptophan-functionalized graphene for sensitive detection of dopamine Applied Surface Science 2015 349 184 189 10.1016/j.apsusc.2015.04.217

[3] Pan N Wang Y Ren X Huang T-S Kim IS Graphene oxide as a polymeric n-halamine carrier and release platform: Highly-efficient, sustained-release antibacterial property and great storage stability Materials Science and Engineering: C 2019 103 109877 10.1016/j.msec.2019.109877 31349493

[4] Pourjavadi A Asgari S Hosseini SH Graphene oxide functionalized with oxygen-rich polymers as a ph-sensitive carrier for co-delivery of hydrophobic and hydrophilic drugs Journal of Drug Delivery Science and Technology 2020 56 101542 10.1016/j.jddst.2020.101542

[5] Ye W Fu J Wang Q Wang C Xue D Electromagnetic wave absorption properties of nicop alloy nanoparticles decorated on reduced graphene oxide nanosheets Journal of Magnetism and Magnetic Materials 2015 395 147 151 10.1016/j.jmmm.2015.07.087

[6] Wang J Ouyang Z Ren Z Li J Zhang P Self-assembled peptide nanofibers on graphene oxide as a novel nanohybrid for biomimetic mineralization of hydroxyapatite Carbon 2015 89 20 30 10.1016/j.carbon.2015.03.024

[7] Kundu N Roy A Banik D Kuchlyan J Sarkar N Graphene oxide and pluronic copolymer aggregates–possible route to modulate the adsorption of fluorophores and imaging of live cells The Journal of Physical Chemistry C 2015 119 44 25023 25035 10.1021/acs.jpcc.5b05251

[8] Zhou T Zhou X Xing D Controlled release of doxorubicin from graphene oxide based charge-reversal nanocarrier Biomaterials 2014 35 13 4185 4194 10.1016/j.biomaterials.2014.01.044 24513318

[9] Bandehali S Moghadassi A Parvizian F Zhang Y Hosseini SM New mixed matrix pei nanofiltration membrane decorated by glycidyl-poss functionalized graphene oxide nanoplates with enhanced separation and antifouling behaviour: Heavy metal ions removal Separation and Purification Technology 2020 242 116745 10.1016/j.seppur.2020.116745

[10] Barrera-Andrade JM Rojas-García E García-Valdés J Valenzuela MA Albiter E Incorporation of amide functional groups to graphene oxide during the photocatalytic degradation of free cyanide Materials Letters 2020 280 128538 10.1016/j.matlet.2020.128538

[11] Ryu SH Sin JH Shanmugharaj AM Study on the effect of hexamethylene diamine functionalized graphene oxide on the curing kinetics of epoxy nanocomposites European Polymer Journal 2014 52 88 97 10.1016/j.eurpolymj.2013.12.014

[12] Ren Q Feng L Fan R Ge X Sun Y Water-dispersible triethylenetetramine-functionalized graphene: Preparation, characterization and application as an amperometric glucose sensor Materials Science and Engineering: C 2016 68 308 316 10.1016/j.msec.2016.05.124 27524025

[13] Kumar H Rajrani Rahul Yadav A Rajni Synthesis, characterization and influence of reduced graphene oxide (rgo) on the performance of mixed metal oxide nano-composite as optoelectronic material and corrosion inhibitor Chemical Data Collections 2020 29 100527 10.1016/j.cdc.2020.100527

[14] Song S Zhai Y Zhang Y Bioinspired graphene oxide/polymer nanocomposite paper with high strength, toughness, and dielectric constant ACS Appl Mater Interfaces 2016 8 45 31264 31272 10.1021/acsami.6b08606 27782385

[15] Fan L Ge H Zou S Xiao Y Wen H Sodium alginate conjugated graphene oxide as a new carrier for drug delivery system International Journal of Biological Macromolecules 2016 93 582 590 10.1016/j.ijbiomac.2016.09.026 27616692

[16] Goenka S Sant V Sant S Graphene-based nanomaterials for drug delivery and tissue engineering Journal of Controlled Release 2014 173 75 88 10.1016/j.jconrel.2013.10.017 24161530

[17] Khatamian M Divband B Farahmand-zahed F Synthesis and characterization of zinc (ii)-loaded zeolite/graphene oxide nanocomposite as a new drug carrier Materials Science and Engineering: C 2016 66 251 258 10.1016/j.msec.2016.04.090 27207061

[18] Zhang L Xia J Zhao Q Liu L Zhang Z Functional graphene oxide as a nanocarrier for controlled loading and targeted delivery of mixed anticancer drugs Small 2010 6 4 537 544 10.1002/smll.200901680 20033930

[19] D’Andrea G Quercetin: A flavonol with multifaceted therapeutic applications? Fitoterapia 2015 106 256 271 10.1016/j.fitote.2015.09.018 26393898

[20] Serban MC Sahebkar A Zanchetti A Mikhailidis DP Howard G Effects of quercetin on blood pressure: A systematic review and meta-analysis of randomized controlled trials Journal of the American Heart Association 2016 5 7 e002713 10.1161/JAHA.115.002713 27405810 PMC5015358

[21] Sahoo PK Pradhan LK Aparna S Agarwal K Banerjee A Quercetin abrogates bisphenol A induced altered neurobehavioral response and oxidative stress in zebrafish by modulating brain antioxidant defence system Environmental Toxicology and Pharmacology 2020 80 103483 10.1016/j.etap.2020.103483 32866630

[22] Wang W Sun C Mao L Ma P Liu F The biological activities, chemical stability, metabolism and delivery systems of quercetin: A review Trends in Food Science & Technology 2016 56 21 38 10.1016/j.tifs.2016.07.004

[23] Vanden Braber NL Paredes AJ Rossi YE Porporatto C Allemandi DA Controlled release and antioxidant activity of chitosan or its glucosamine water-soluble derivative microcapsules loaded with quercetin International Journal of Biological Macromolecules 2018 112 399 404 10.1016/j.ijbiomac.2018.01.085 29421395

[24] Kumari A Yadav SK Pakade YB Singh B Yadav SC Development of biodegradable nanoparticles for delivery of quercetin Colloids Surf B Biointerfaces 2010 80 2 184 192 10.1016/j.colsurfb.2010.06.002 20598513

[25] Cai X Fang Z Dou J Yu A Zhai G Bioavailability of quercetin: Problems and promises Curr Med Chem 2013 20 20 2572 2582 10.2174/09298673113209990120 23514412

[26] Trendafilova I Szegedi A Mihály J Momekov G Lihareva N Preparation of efficient quercetin delivery system on zn-modified mesoporous sba-15 silica carrier Materials Science and Engineering: C 2017 73 285 292 10.1016/j.msec.2016.12.063 28183610

[27] Marcano DC Kosynkin DV Berlin JM Sinitskii A Sun Z Improved synthesis of graphene oxide ACS Nano 2010 4 8 4806 4814 10.1021/nn1006368 20731455

[28] Khaligh NG Ghasem-Abadi PG N-sulfonic acid poly (4-vinylpyridinum) hydrogen sulfate as a novel, efficient, and reusable solid acid catalyst for acylation under solvent-free conditions Chinese Journal of Catalysis 2014 35 7 1126 1135 10.1016/S1872-2067(14)60052-8

[29] Khaligh NG Preparation, characterization and use of 3-methyl-1-sulfonic acid imidazolium hydrogen sulfate as an eco-benign, efficient and reusable ionic liquid catalyst for the chemoselective trimethylsilyl protection of hydroxyl groups Journal of Molecular Catalysis A: Chemical 2011 349 1–2 63 70 10.1016/j.molcata.2011.08.021

[30] Oger N Lin YF Labrugère C Le Grognec E Rataboul F Practical and scalable synthesis of sulfonated graphene Carbon 2016 96 342 350 10.1016/j.carbon.2015.09.082

[31] Liu J Xue Y Dai L Sulfated graphene oxide as a hole-extraction layer in high-performance polymer solar cells The Journal of Physical Chemistry Letters 2012 3 14 1928 1933 10.1021/jz300723h 26292015

[32] Zhao G Jiang L He Y Li J Dong H Sulfonated graphene for persistent aromatic pollutant management Advanced Materials 2011 23 34 3959 3963 10.1002/adma.201101007 21800380

[33] Yang A Li J Zhang C Zhang W Ma N One-step amine modification of graphene oxide to get a green trifunctional metal-free catalyst Applied Surface Science 2015 346 443 450 10.1016/j.apsusc.2015.04.033

[34] Liu Y Xu L Liu J Liu X Chen C Graphene oxides cross-linked with hyperbranched polyethylenimines: Preparation, characterization and their potential as recyclable and highly efficient adsorption materials for lead(ii) ions Chemical Engineering Journal 2016 285 698 708 10.1016/j.cej.2015.10.047

[35] Zhang W Wang S Ji J Li Y Zhang G Primary and tertiary amines bifunctional graphene oxide for cooperative catalysis Nanoscale 2013 5 13 6030 6033 10.1039/C3NR01323E 23714770

[36] Navaee A Salimi A Efficient amine functionalization of graphene oxide through the bucherer reaction: An extraordinary metal-free electrocatalyst for the oxygen reduction reaction RSC Advances 2015 5 74 59874 59880 10.1039/C5RA07892J

[37] He D Kou Z Xiong Y Cheng K Chen X Simultaneous sulfonation and reduction of graphene oxide as highly efficient supports for metal nanocatalysts Carbon 2014 66 312 319 10.1016/j.carbon.2013.09.005

[38] Kim NH Kuila T Lee JH Simultaneous reduction, functionalization and stitching of graphene oxide with ethylenediamine for composites application Journal of Materials Chemistry A 2013 1 4 1349 1358 10.1039/C2TA00853J

[39] Park O-K Hahm MG Lee S Joh H-I Na S-I In situ synthesis of thermochemically reduced graphene oxide conducting nanocomposites Nano Letters 2012 12 4 1789 1793 10.1021/nl203803d 22260510

[40] Ferrari AC Meyer JC Scardaci V Casiraghi C Lazzeri M Raman spectrum of graphene and graphene layers Physical Review Letters 2006 97 18 187401 10.1103/PhysRevLett.97.187401 17155573

[41] Park O-K Lee S Joh H-I Kim JK Kang P-H Effect of functional groups of carbon nanotubes on the cyclization mechanism of polyacrylonitrile (pan) Polymer 2012 53 11 2168 2174 10.1016/j.polymer.2012.03.031

